# Quality of life in climacteric women assisted by primary health care

**DOI:** 10.1371/journal.pone.0211617

**Published:** 2019-02-27

**Authors:** Geraldo Edson Souza Guerra, Antônio Prates Caldeira, Fernanda Piana Santos Lima de Oliveira, Maria Fernanda Santos Figueiredo Brito, Kelma Dayana de Oliveira Silva Gerra, Carlos Eduardo Mendes D’Angelis, Luís Antônio Nogueira dos Santos, Lucineia de Pinho, Josiane Santos Brant Rocha, Daniela Araújo Veloso Popoff

**Affiliations:** 1 Postgraduate Program in Primary Health Care, State University of Montes Claros, Montes Claros, Minas Gerais, Brazil; 2 Fipmoc University Center (UNIFIPMoc), Montes Claros, Minas Gerais, Brazil; Charite Medical University Berlin, GERMANY

## Abstract

This cross-sectional study evaluated the quality of life and associated factors of climacteric women in Brazil using a random and representative sample of women assisted by primary care professionals. We investigated the variables using the Menopause-Specific Quality of Life Questionnaire, MENQOL, whose mean scores were compared using Mann–Whitney and Kruskal–Wallis tests according to the sample characteristics. The variables associated with the outcomes in univariate analyses with a p≤0.2 were jointly evaluated using multiple linear regression. In this study, 849 women ranging in age from 40 to 65 years were evaluated. The predictors of poor quality of life in the vasomotor domain were women with severe climacteric symptoms (p<0.001), increased *Body Mass Index* (BMI) (p = 0.006), sleep (p = 0.022), and postmenopausal (p<0.001) alterations. For the psychosocial domain, the associated variables were severe climacteric symptoms (p<0.001) and sleep alterations (p<0.001); for the physical domain, the associated variables were severe climacteric symptoms (p<0.001), increased BMI (p<0.001), sleep (p<0.001), and postmenopausal (p<0.001) alterations. Severe climacteric symptoms, low sleep quality, increased BMI, and postmenopausal status were factors that were more associated with impairments in quality of life. With the increase in life expectancy, we suggest that greater attention should be paid to women’s quality of life associated with climacteric symptoms.

## Introduction

Brazil is experiencing a profound change in its age structure, with clear population aging and an increase in the number of women [1]. Earlier, the World Health Organization (WHO) estimated a life expectancy of approximately 78 years in developing countries by 2015, which would have an impact on the number of climacteric women [2]. Considering this perspective, women will be the main users of the Brazilian public health services.

The climacteric phase is marked by the decline of estrogen production, which, by participating in several biological processes, may involve cardiovascular, cerebral, cutaneous, genitourinary, bony and vasomotor changes, as well as changes in mood and appetite [3]. The most prevalent symptoms in this phase are night sweats, hot flashes, vaginal dryness, muscular flaccidity of the pelvic floor, dyspareunia, and insomnia [3–5]. The physical changes that occur with the aging process may also lead to changes in one’s self-image [6]. Anxiety and depression reports demonstrate alterations in neurotransmission activity as possible reflexes of the decreased hormonal levels [7].

In addition to the common alterations that women face in this phase due to the hypoestrogenism, the climacteric experience is unique to each woman and can be influenced by hereditary, social, cultural, and lifestyle factors. Furthermore, many women experience physical and mental changes that are often associated with aging. This process may influence women’s quality of life, which is of considerable social and scientific relevance [8]. In recent decades, the need to evaluate quality of life has been perceived, due in part to the new paradigms that influence public policies and assistance practices. Thanks to these changes, quality of life improvement has become one of the expected results in the fields of health promotion and disease prevention [9].

A review study about climacteric women’s quality of life reported nine instruments that have been most used in scientific studies. The measures used in the studies vary in the type and number of domains that are reported, including psychosocial, somatic, vasomotor, urogenital, and sexual domains, as well as cognition, sleep, appetite occupation, and quality of life [10]. Among the available instruments, the *Menopause-specific Quality of Life Questionnaire* (MENQOL) seems to be better compared to the preexisting instruments, as it includes several aspects of the *Kupperman Index*, which monitors the effects of treatments instituted in the climacteric and the General Welfare Scale [11]. Although widely used throughout the world, MENQOL is rarely used in Latin America and Brazil [12].

In the last years, Brazil has experienced a great expansion of the coverage of Primary Health Care services through the implantation of multiprofessional health teams, strategically located in peripheral areas of the cities or areas of greater social needs. This proposal, known as the Family Health Strategy, includes at least one doctor, one nurse, one nursing assistant and four to six full-time community health workers and attends an average of 800 to 1,000 families. However, there are still few studies on the female population assisted and monitored by these teams [13].

Although there are many studies about quality of life in different groups, research related to the menopausal transition is limited, and the factors associated with loss of quality of life in climacteric women are still not conclusive. Considering the gaps identified in the literature, this study aimed to evaluate the quality of life of climacteric women assisted by primary healthcare professionals, seeking to identify factors associated with the worst scores of quality of life, according to MENQOL.

## Materials and methods

This is a cross-sectional analytical study conducted between August 2014 and August 2015. The target population was composed of women ranging in age from 40 to 65 years (climacteric period), enrolled in the 73 Basic Health Units (BHUs) in a medium-sized city in the northern region of Minas Gerais, Brazil.

**Fig 1:**
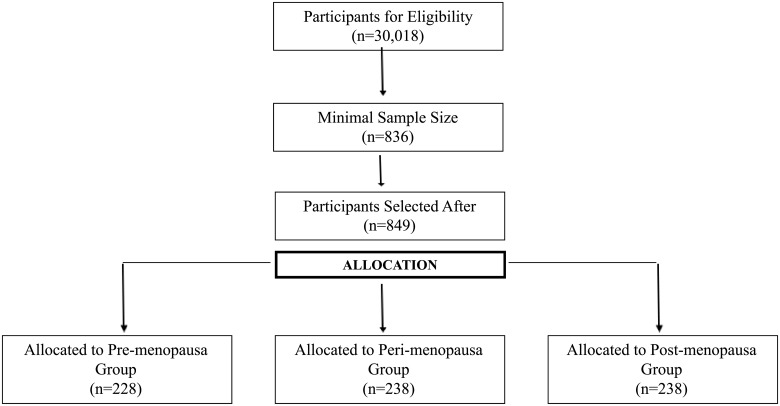
Flowchart of selection process, 2015.

The sampling was probabilistic, with the participants selected according to the lottery method, following a sampling plan in two stages: (1) conglomerate, assuming each BHU as a sample unit, and (2) stratified random selection, according to the climacteric period (pre, peri-, and postmenopausal) among all women included in the study in each BHU. When the selected women were not found, a new lottery was performed until the pre-established number was achieved, according to the proportional sharing. The sample size was calculated based on the total number of women in the age group enrolled in the BHU (n = 30,018), considering a 95% confidence level and a sampling error of 5%. As there were no previous studies of this subject in the region, a 50% prevalence was estimated for the measure/instrument being studied. Considering the conglomerate method used for sampling, the sample number identified was multiplied by a correction factor equal to 2 plus 10% for eventual losses. Thus, the minimal number of women being evaluated was 836. The exclusion criteria were pregnant women, those who had recently given birth, were bedridden, and/or exhibited cognitive impairments, as well as women who reported bilateral oophorectomy with or without hysterectomy. The flow of study participants was demonstrated in a flowchart (Fig 1).

The researchers conducted prior training with all collectors/interviewers through meetings. At the first moment, the validated questionnaires were presented, punctuating the validation characteristics; later, an in-depth reading was performed by the collectors/interviewers to elucidate the doubts; and at the end of this stage, the questionnaires were applied among the interviewers. The responsible researchers supervised the pilot study and the data collection. Therefore, a pilot study was performed using a previous data collection to enable the team and to set possible adjustments in the collection instruments.

The women selected for this and subsequent phases of this study were invited by the community health agent to visit the BHU at a predetermined date and time, where the research team was able to apply all of the instruments of data collection. The data collection instrument included sociodemographic data, life habits, body composition, clinical aspects, and morbidities present. The quality of life was assessed using MENQOL, a self-administered instrument consisting of 29 Likert scale items that evaluate the menopausal symptoms in the last month, distributed in four domains: vasomotor (items 1–3), psychosocial (items 4–10), physical (items 11–26), and sexual (items 27–29). The items were classified as being absent “1” or present “2”: if present, the discomfort was classified on a scale from zero (no discomfort) to 6 (extremely uncomfortable) in this domain [11,14]. For each item, the score would range from 1 (not present) to 8 (maximum bother). The mean was calculated for each subscale by dividing the sum of the domain items by the number of items in the domain.

The mean scores for each MENQOL domain were compared for the following demographic and socioeconomic variables: age (40–65 years; 46–51 years; 52–65 years), race/skin color (black vs. other), marital status (married or stable union vs. single/divorced/widowed), family income (below a minimum wage vs. above a minimum wage), and schooling (elementary school I, elementary school II, high school, or graduate school). Furthermore, the mean scores were compared for the following clinical variables: climacteric classification (premenopausal, peri-menopausal, and postmenopausal); magnitude of climacteric symptomatology (mild, moderate, severe), *Body Mass Index* (BMI) (adequate weight; overweight; obesity), physical activity, assessed by the *International Physical Activity Questionnaire* (IPAQ) (very active or active; irregularly active or sedentary) [15], sleep quality, according to the *Pittsburgh Sleep Quality Index* (PSQI) (loss of sleep quality vs. good sleep quality) [16], and records of arterial hypertension (yes vs. no) and diabetes (yes vs. no).

Women with a habitual menstrual cycle were classified as premenopausal; those with an irregular menstrual cycle, varying from 2 to 11 months, were classified as peri-menopausal; and women with an interrupted menstrual cycle for more 12 months were classified in the postmenopausal phase.

Information on the climacteric symptomatology was obtained using the *Kupperman Index*, an instrument adapted and validated for research and clinical practice purposes to monitor the treatments instituted in climacteric persons [17]. The answers are based on the following scores: 0 (no symptoms), 1 (mild symptoms), 2 (moderate symptoms), and 3 (severe symptoms). For the total scores calculated, the symptoms studied presented differential weights, in which hot flashes assumed more relevance (weight 4); paresthesia, insomnia, and nervousness received an intermediate value (weight 2); and symptoms such as sadness, dizziness, weakness, arthralgia/myalgia, headache, palpitation, and tingling received weight 1. Multiplying the intensity of symptoms by the respective correction factor followed by the sum of results obtained, the climacteric syndrome may be classified as mild, moderate, severe or without information. This is standard procedure for the MENQOL.

BMI was obtained by dividing body weight by squared height (P/E2). The results of the BMI were classified according to the following criteria: adequate weight (18.5–24.9); overweight (25.0–29.9), and obesity (≥30). The height was measured using a SECA 206 (Seca GmbH & Co.–Germany) anthropometer in a wall with 90° in relation to the floor and without skirting boards with the woman in a proper position to evaluate the data. To evaluate the weight (kg), a portable OMEGA 870 digital scale was used, and the women wore light clothing.

Regarding physical activity, the IPAQ, developed and validated by Craig et al. (for the population ranging in age from 18 to 65 years) was validated for Portuguese by Matsudo et al. [15]. The abbreviated version adopted in this review comprises six questions regarding physical activity performed in the last week, for at least 10 continuous minutes, before the questionnaire commenced. According to the instrument, the following classification is adopted: very active, active, irregularly active or sedentary.

The PSQI is a self-report questionnaire, validated in Brazil, that evaluates sleep quality over the last 4 weeks and distinguishes “poor” from “good” sleep quality [16]. It consists of 19 graduated questions (Likert scale), where a score of zero points means no difficulty and a score of 3 points means severe difficulties. The scores are measured for seven components: (1) subjective sleep quality; (2) sleep latency; (3) sleep duration (4) habitual sleep efficiency; (5) sleep disturbances; (6) use of sleep medications; and (7) daytime dysfunction. The sum of scores (from these seven components) varies from 0 to 20. Scores from 0 to 5 indicate good sleep quality, and scores from 6 to 20 indicate loss of sleep quality.

Records of high blood pressure and diabetes were self-reported, considering the answer to the question: “Has any doctor ever told you that you have high blood pressure/diabetes?”

The data were analyzed using the *Statistical Package for the Social Sciences* (SPSS) program, version 21.0, by descriptive statistics, with absolute and relative frequencies, means and standard deviations. The mean scores for each MENQOL domain were compared using the Mann–Whitney (two categories) and Kruskal–Wallis (three or more categories) tests, according to the demographic, socioeconomic, and clinical characteristics of the women participating in the study. The variables associated with the outcomes in the univariate analyses with p≤0.2 were jointly evaluated using multiple linear regression, in which the significance level was 5% (p<0.05).

All study participants signed a Free and Informed Consent Term, and the Research Ethics Committee of the Faculdades Integradas Pitágoras approved the research project (Protocol: 817.166).

## Results

In this study, 849 climacteric women participated, of whom the majority ranged in age from 52 to 65 years (45.0%), were married or in a stable union (62.7%), with schooling periods below 9 years (41.0%). Approximately 44% of the women reported a family income that was below minimum wage. Table 1 presents the main demographic and socioeconomic characteristics of the study population.

**Table 1:** Demographic and socioeconomic characteristics of climacteric women assisted by primary health care, 2015.

Variable	n	%
Age		
40–45 years	228	27.7
46–51 years	238	27.3
52–65 years	383	45.0
Skin color		
Brown	535	63.6
Black	109	12.6
White	152	17.4
Yellow	38	4.5
Indigenous	9	1.2
No information	6	0.8
Marital status		
Single	82	9.6
Married/stable union	542	62.7
Divorced	133	16.7
Widowed	90	10.6
No information	2	0.3
Religion		
Catholic	568	66.9
Evangelical	263	31.0
Other/no information	18	2.1
Schooling		
Graduate school/high school	276	32.0
Elementary school II	226	26.7
Elementary school I	344	41.0
No information	3	0.3
Family income		
Above minimum wage	471	55.6
Below minimum wage	378	44.4
Work outside the home		
Yes	342	40.6
No	500	58.7
No information	7	0.7

Table 2 describes the participants’ clinical characteristics, life habits, and health care. Most women were in the postmenopausal phase (43.0%), overweight (73.4%) and irregularly active (55.5%). The most prevalent morbidity was arterial hypertension (46.0%).

**Table 2:** Characteristics related to life habits and health care of climacteric women assisted by primary health care, 2015.

Variable	n	%
Climacteric classification		
Premenopausal	230	26.9
Peri-menopausal	263	29.4
Postmenopausal	356	43.7
Climacteric symptomatology		
Mild	524	62.0
Moderate	241	28.2
Severe	83	9.7
No information	1	0.1
BMI		
Adequate weight	223	25.8
Overweight	323	37.5
Obesity	298	35.9
No information	5	0.8
Arterial hypertension		
Yes	386	46.0
No	406	48.8
No information	57	5.3
Diabetes		
Yes	115	14.1
No	680	81.2
No information	54	4.7
IPAQ		
Very active/active	109	12.8
Irregularly active	471	55.5
Sedentary	269	31.7
PSQI		
No disturbance	271	31.2
With disturbance	529	63.1
No information	49	5.7

Regarding the demographic and socioeconomic characteristics, the mean scores obtained for each MENQOL domain are described in Table 3. Concerning age, the mean scores were higher for the age range from 52 to 65 years for the vasomotor, physical, and sexual domains. No differences were found between black and nonblack women or between family income categories.

**Table 3:** Demographic and socioeconomic characteristics of climacteric women assisted by primary health care according to MENQOL domains, 2015.

Characteristics	n	%	Vasomotor			Psychosocial			Physical			Sexual		
			Mean	SD	p	Mean	SD	p	Mean	SD	p	Mean	SD	p
**Age**					<0.001			0.439			0.033			<0.001
40–45	228	27.7	3.01	2.38		3.66	1.75		3.52	1.56		2.80	1.44	
46–51	238	27.3	3.93	2.62		3.67	1.74		3.83	1.61		3.09	1.48	
52–65	383	45.0	4.11	2.71		3.55	1.82		3.94	1.68		3.66	1.71	
**Skin color**					0.297			0.830			0.646			0.062
Black	644	76.3	3.81	2.65		3.58	1.78		3.79	1.65		3.24	1.59	
Nonblack	200	23.7	3.64	2.59		3.73	1.75		3.85	1.60		3.38	1.77	
**Marital status**					0.108			<0.001			<0.001			0.167
Married/stable union	542	64	3.74	2.60		3.51	1.68		3.76	1.56		3.24	1.59	
Single/divorced/widow	305	36	3.81	2.70		3.80	1.94		3.89	1.77		3.33	1.70	
**Family income**					0.674			0.871			0.608			0.420
Above minimum wage	471	55.5	3.71	2.59		3.58	1.69		3.82	1.56		3.23	1.63	
Below minimum wage	378	44.5	3.83	2.70		3.66	1.88		3.79	1.73		3.31	1.63	
**Schooling**					<0.001			0.151			0.240			<0.001
High school or graduate school	276	32.0	3.23	2.48		3.42	1.68		3.68	1.56		2.95	1.53	
Elementary school II	226	27.0	3.96	2.66		3.72	1.86		3.79	1.66		3.22	1.56	
Elementary school I	344	41.0	4.06	2.67		3.70	1.78		3.92	1.68		3.57	1.70	

According to Table 4, which presents the scores obtained for each MENQOL domain according to the clinical characteristics, women with severe climacteric symptomatology presented higher scores in all domains. Women classified as obese and those with poor sleep quality also presented higher scores in all domains.

**Table 4:** Life and health habits of climacteric women assisted by primary health care according to MENQOL domain, 2015.

Characteristics	n	%	Vasomotor			Psychosocial			Physical			Sexual		
			Mean	SD	p	Mean	SD	p	Mean	SD	p	Mean	SD	p
**Climacteric classification**					<0.001			0.173			0.006			<0.001
Premenopausal	226	27.0	2.98	2.37		3.65	1.75		3.57	1.57		2.79	1.45	
Peri-menopausal	238	28.4	3.93	2.62		3.67	1.74		3.83	1.61		3.09	1.48	
Postmenopausal	373	44.6	4.14	2.71		3.56	1.83		3.94	1.70		3.66	1.71	
**Climacteric symptomatology**					<0.001			<0.001			<0.001			<0.001
Severe	83	9.8	6.92	1.89		5.58	1.63		5.89	1.24		4.87	1.58	
Moderate	241	28.2	4.83	2.49		4.33	1.67		4.40	1.48		3.62	1.63	
Mild	524	62.0	2.77	2.18		2.98	1.47		3.21	1.37		2.85	1.43	
**BMI**					<0.001			0.004			<0.001			0.056
Adequate weight	223	26.4	3.28	2.48		3.64	1.77		3.49	1.55		3.11	1.62	
Overweight	323	38.3	3.68	2.62		3.34	1.75		3.67	1.59		3.21	1.56	
Obesity	298	35.3	4.21	2.68		3.84	1.79		4.20	1.67		3.46	1.69	
**Arterial hypertension**					<0.001			0.059			<0.001			0.002
No	406	51.3	3.43	2.50		3.52	1.71		3.65	1.61		3.10	1.54	
Yes	386	48.7	4.30	2.76		3.80	1.87		4.08	1.64		3.48	1.70	
**Diabetes**					<0.001			0.009			0.001			0.038
No	680	85.5	3.68	2.59		3.58	1.75		3.77	1.61		3.23	1.59	
Yes	115	14.5	4.90	2.84		4.08	1.96		4.34	1.73		3.54	1.83	
**Physical activity**					0.093			0.021			0.358			0.015
Very active/active	109	12.8	4.00	2.55		3.96	1.67		3.94	1.62		3.49	1.51	
Irregularly active	471	55.5	3.65	2.64		3.48	1.74		3.74	1.61		3.14	1.62	
Sedentary	269	31.7	3.86	2.65		3.71	1.87		3.88	1.69		3.41	1.67	
**PSQI**					<0.001			<0.001			<0.001			<0.001
With disturbance	529	66.1	4.27	2.67		4.00	1.74		4.25	1.59		3.56	1.66	
No disturbance	271	33.9	2.87	2.35		2.85	1.55		2.98	1.38		2.70	1.37	

All variables that were associated with the outcome in univariate analyses with p≤0.2 were globally evaluated using multiple linear regression (Table 5), with each of the MENQOL domains as dependent variables. The intensity predictors found to be most elevated in the vasomotor domain were women with severe climacteric symptoms (p<0.001), increased BMI (p = 0.006), disturbance in sleep quality (p = 0.022), and in the postmenopausal phase (p<0.001). In the psychosocial domain, the associated variables were severe climacteric symptoms (p<0.001) and sleep quality alterations (p<0.001). In the sexual domain, the associated variables were as follows: intense climacteric symptoms (p<0.001), sleep quality alterations (p<0.001), and more advanced age (p<0.001).

**Table 5:** Linear regression according to the demographic and socioeconomic variables, life habits, and health care of climacteric women in relation to the MENQOL domains, 2015.

Domain	Variable	β	Confidence Interval (95%)	p	R^2^
**Vasomotor**					0.339
	Climacteric symptomatology	-0.490	-2.174 to -1.677	<0.001	
	BMI	-0.084	-0.497 to -0.083	0.006	
	PSQI	-0.073	-0.767 to -0.059	0.022	
	Climacteric classification	-0.161	-0.711 to -0.325	<0.001	
**Psychosocial**					0.261
	Climacteric symptomatology	-0.435	-1.311 to -0.967	<0.001	
	PSQI	-0.164	-0.871 to -0.374	<0.001	
**Physical**					0.333
	Climacteric symptomatology	-0.440	-1.213 to -0.912	<0.001	
	BMI	-0.150	-0.444 to -0.194	<0.001	
	PSQI	-0.216	-0.970 to -0.535	<0.001	
**Sexual**					0.185
	Climacteric symptomatology	-0.303	-0.889 to -0.557	<0.001	
	PSQI	-0.143	-0.732 to -0.255	<0.001	
	Age	-0.145	-0.416 to -0.148	<0.001	

## Discussion

The present study evaluated the quality of life of climacteric women assisted by the Family Health Strategy teams, registering significantly impaired quality of life, associated with greater intensities of climacteric symptoms, poor sleep quality, increased BMI, and advanced age in different domains of the instrument used. Although the national literature has studies evaluating the quality of life of climacteric women [18–20], records of the MENQOL were not observed, which is indicated as one of the best specific instruments for evaluating the quality of life in this female-relevant phase [10–12,14]. The climacteric represents a phase of important hormonal and social changes in women. As life expectancy increases, the exposure to the consequences of this condition becomes greater, and the need for greater investment in improving the quality of life of climacteric women becomes more critical.

The transition from the fertility period to the ovarian dysfunction period is a gradual and relatively complex process. The literature registers considerable variances in the prevalence and symptoms patterns in different population studies, likely due to the cultural diversity, precepts and traditions, diet, and other factors related to lifestyle, thereby justifying the need for regional studies [21–24].

In the present study, the higher scores in the instrument, which reflect poor quality of life, were more commonly observed in the physical and vasomotor domains compared to the psychosocial and sexual domains, corroborating the findings of previous studies [21–27]. This finding demonstrates the relevance of vasomotor and physical (pain, fatigue, weight gain, etc.) symptoms during the climacteric phase and the need to further evaluate and comprehend these symptoms [5].

More intense climacteric symptoms, as assessed using the *Kupperman Index*, and the loss of sleep quality were observed as variables associated with the greater quality of life impairment in all MENQOL domains, even after multiple analyses. Regarding the climacteric symptomatology, other studies have shown similar findings [21–28].

This association shows, on the one hand, the intimate relationship between the two instruments and, on the other hand, the intrinsic relationship between the dimensions measured for the quality of life and the climacteric symptoms, which go beyond the vasomotor physiological aspects and reach physical, psychological, and sexual aspects [5].

Concerning sleep quality, it is comprehensible that hot flashes and night sweats, common symptoms during menopause, present direct interference on sleep. Several studies have reported that such manifestations may effectively impair the duration and quality of sleep, resulting in fatigue, irritability, forgetfulness, acute physical discomfort, and decreases in labor productivity and general quality of life [5,29,30].

The main findings of this study also demonstrate an independent and statistically significant association between increased BMI and worse quality of life scores in the vasomotor and physical domains. Such results are consistent with the results of studies performed in other countries [21,27,30,31–33]. Women in these conditions are not only more prone to experience vasomotor symptoms but also feel more uncomfortable because of these symptoms. A likely explanation for these findings would be that increased waist circumference and increased BMI are important indicators of physical difficulties with basic activities of daily living [32].

In this study, women with more advanced age and in the postmenopausal phase presented the worst scores for quality of life in the sexual and vasomotor domains, respectively. These findings are coincident with two studies [24,34]. However, the literature is inconsistent regarding the theme, and other authors have observed that the incidence of vasomotor problems tends to be higher during the peri-menopausal phase, appearing to decrease with age, which, according to these authors, demonstrates a greater capacity for women to address climacteric symptoms [26,27].

Although other studies that used MENQOL have identified a quality of life impairment in association with social and economic variables [31,35], the present study did not report any association regarding these variables. This lack of association probably stems from the similar economic context of all women evaluated (women assisted by public health service teams). [36]

The delimitation of the target population is one of the limitations of the study. It is not possible to make inferences for the entire population, considering that only women assisted by *Family Health Strategy* (FHS) teams were evaluated. However, it should be noted that researchers used this strategy to achieve greater adherence to the study. The fact that the instrument evaluates symptoms experienced in the last 4 weeks may be linked to memory bias and even to memory limitations in older women. Additionally, because we conducted a cross-sectional study, it is not possible to establish causal associations and thus evaluate the factors that impact quality of life over time for the population studied. Furthermore, the instrument that was originally developed for evaluating menopausal women was not validated for the pre- and peri-menopausal periods. However, in recent studies that use MENQOL, there is a trend that women in previous stages also experience signs and symptoms classically attributed to menopause [12,37,38].

Another limitation that needs to be addressed refers to the no report of the use of hormone therapy or other treatments by the participants, which did not allow us to capture participants who were taking the treatments. There have been no reports of the use of hormone replacement therapy (HRT) or other treatments for menopause, perhaps because it is a population in need that requires assistance through the country’s public services, which does not offer this type of therapy or treatment. Unfortunately, we did not investigate the use of alternative treatments, such as herbal medicines, which is quite common in this population.

Despite the limitations identified, the use of the MENQOL, which has been successfully used throughout the world but is not widely adopted in Brazil, is an important aspect of the study, which had a satisfactory sample number and evaluated a population group frequently excluded from public health policies. It should be emphasized that many of the aspects addressed in the assessment of quality of life involve the presence of signs and symptoms that should be addressed by health professionals, seeking to understand their influence on everyday relationships and alleviate, whenever possible, the discomforts reported by women. In this sense, the present study warns health professionals to evaluate climacteric women more carefully and judiciously, stimulating and promoting healthy lifestyles, valuing subjective aspects and appropriate and timely clinical manifestations, as a lack of information may restrict awareness of health choices, especially for less-educated women [39]. These recommendations are particularly important considering the prospect of a large increase in the elderly female population in the near future, which will represent the main group of health service users.
